# A fast kernel independence test for cluster-correlated data

**DOI:** 10.1038/s41598-022-26278-9

**Published:** 2022-12-15

**Authors:** Hoseung Song, Hongjiao Liu, Michael C. Wu

**Affiliations:** 1 Public Health Sciences Division, Fred Hutchinson Cancer Research Center, Seattle, WA 98109 USA; 2 Department of Biostatistics, University of Washington, Seattle, WA 98195 USA

**Keywords:** Statistics, Statistical methods

## Abstract

Cluster-correlated data receives a lot of attention in biomedical and longitudinal studies and it is of interest to assess the generalized dependence between two multivariate variables under the cluster-correlated structure. The Hilbert–Schmidt independence criterion (HSIC) is a powerful kernel-based test statistic that captures various dependence between two random vectors and can be applied to an arbitrary non-Euclidean domain. However, the existing HSIC is not directly applicable to cluster-correlated data. Therefore, we propose a HSIC-based test of independence for cluster-correlated data. The new test statistic combines kernel information so that the dependence structure in each cluster is fully considered and exhibits good performance under high dimensions. Moreover, a rapid *p* value approximation makes the new test fast applicable to large datasets. Numerical studies show that the new approach performs well in both synthetic and real world data.

## Introduction

Measuring general, possibly nonlinear, dependence between two multivariate variables plays a significant role in many scientific applications. For example, assessing the relationship between the overall composition of the microbiome, which includes hundreds of microbial taxa, and various host metabolites from a specific metabolic pathway is often of central interest in many studies^1–3^. Determining and understanding the dependence between such variables have successfully provided important clues as to the mechanisms and biological interactions among the variables leading to better understanding of the systems underlying many different conditions.

In the meantime, correlated observations are also frequently obtained in many practical situations. Family-based samples in genome-wide association studies are often used to assess a genetic association to a disease^4^. Repeated/longitudinal observations are also prevalent in biomedical research and the goal of research is to figure out how explanatory variables influence an outcome over time^5^. Within this context of cluster-correlated data, there is also pressing interest in understanding the general dependency between multivariate variables, e.g. the correlation between microbiome composition and metabolic pathways, in longitudinally collected samples.

An example of a study in which we are interested in multivariate dependency in longitudinal samples which also motivates this project is the Menopause Studies—Finding Lasting Answers for Symptoms and Health (MsFLASH) study. MsFLASH was a randomized clinical trial in which women were randomized to one of three arms (two placebo, and one experimental treatment with vaginal estrogen) with the objective of improving symptoms of menopause^6^. The underlying biological hypothesis was that the introduction of estrogen into the vaginal environment would shift the microbiota and result in reduced symptoms. However, despite considerable preliminary research and years of effort, the trial was null and no differences in symptom improvement were identified across the arms. Thus, in a post mortem evaluation of the trial, investigators have been studying why the trial failed despite the preliminary data, including evaluations of whether the underlying hypotheses were correct. Initial work concentrated on the microbiome and they were able to show that, in fact, the microbiome was altered by the introduction of estrogen. Now, a subsequent analysis is focused on whether these microbes are associated with metabolic activity, as one would expected. Ultimately, metabolites are the biochemicals produced by the microbes that should impact symptom development. Thus, a central analytic objective was to evaluate the general dependency between microbiome composition and pre-specified metabolic pathways across time, yet how to optimally conduct this analysis is unclear.

Classical measures of association, such as Pearson correlation^7^, Kendall’s $\tau$^8^, and Spearman’s $\rho$^9^, are mainly focused on a simple dependence structure and they could be zero even when two random variables are dependent. As we are entering the big data era, challenging data, both in the dimension and size, is becoming prevalent, and the attention to the association testing method for detecting complex dependence structures is also naturally increasing. Hence, many methods have been proposed to develop tests of independence against general types of alternatives, such as the RV coefficient or its extensions^10–13^, the distance covariance coefficient or its extensions^14–16^, the graph-based test^17^, the rank-based test^18,19^, and the kernel-based test^20,21^.

In particular, kernel-based tests are often utilized to evaluate the association between overall microbiome compositions and outcomes of interest or host gene expressions^22–24^. It is well known that kernels can be used to embed the microbiome structure and many different kernels have been developed: UniFrac kernels can accommodate the phylogenetic structure^25^, generalized UniFrac kernels are sensitive to abundance changes in moderately abundant lineages^26^, and the Bray–Curtis kernel quantifies the taxonomic dissimilarity of two microbial communities^22^.

In this paper, we base our approach on the most popular kernel-based test, the Hilbert–Schmidt independence criterion (HSIC), proposed by Gretton et al.^21^. As a nonparametric approach, the HSIC has the potential to capture arbitrary dependence between two random variables. It can be viewed as the distance between the joint distribution and the product of the marginals embedded in a reproducing kernel Hilbert space (RKHS).

However, most kernel-based methods assume that pairs are independent and identically distributed (i.i.d.) and they thus cannot be directly applied to correlated data, particularly clustered data. Moreover, the asymptotic distribution of the HSIC to obtain the threshold of the test given level $\alpha$ of the test is not practical since the null distribution has a complicated form, and cannot be evaluated directly. Therefore, a permutation test is usually preferred in many applications, however, it is computationally prohibitive when the sample size is large or when the alpha level is low, as in the case of our motivating data where we are interested in studying associations between the microbiome and numerous metabolic pathways.

Based on the HSIC, we propose a new test of independence for cluster-correlated data. The new test combines kernel information so that the dependence structure in each cluster is fully considered. Furthermore, compared to other HSIC-based tests that rely on costly Monte Carlo permutation procedures, a closed form of *p* value approximation is proposed, making the new test much faster and more efficient than the existing tests, particularly for large samples. Numerical studies demonstrate that the new method is powerful under high dimensions in both synthetic and real world data. Our work is related to recent work of Zhan et al.^13^, but differs in that the new test statistic has a computationally more efficient form.

The organization of the paper is as follows. In “Materials and methods” section, we provide our problem setting on clustered data and briefly review the test based on the HSIC. We then propose the new test statistic and the testing procedure for cluster-correlated data. In “Results” section, we examines the performance of the new tests under various simulation settings and the new approach is illustrated by a real data application on vaginal microbiome data. Finally, the discussion is given in “Discussion” section.

## Materials and methods

### Problem setting

The goal is to test for association between two sets of variables *X* and *Y*, such as microbiome composition, gene expression, or profiles of other types of genomic data. Specifically, let *X* and *Y* be multivariate random variables with marginal distributions $f_{X}$ on *X* in $\mathcal {R}^{p}$ and $f_{Y}$ on *Y* in $\mathcal {R}^{q}$, respectively. Let $f_{XY}$ be the joint distribution on $X\times Y$. Then, we aim to test

1 $$\begin{aligned} \text {H}_{0}: f_{XY} = f_{X}f_{Y} \ \ \text {versus} \ \ \text {H}_{1}: f_{XY} \ne f_{X}f_{Y}. \end{aligned}$$

We consider samples of clustered data: observations $\left( X_{1},Y_{1}\right) , \ldots , \left( X_{N},Y_{N}\right) \in \left( X, Y\right)$ of total sample size *N* are drawn identically from $f_{XY}$ and can be divided into *m* clusters of size *l* $(i = 1, \ldots , m)$, that is,

2 $$\begin{aligned} \left\{ \left( X_{1}^{(i)},Y_{1}^{(i)}\right) , \ldots , \left( X_{l}^{(i)},Y_{l}^{(i)}\right) \right\} _{i=1}^{m}. \end{aligned}$$

Here, $ml = N$ and *m* clusters are independent from each other while having identical within-cluster correlation structure.

### Hilbert–Schmidt independence criterion

The Hilbert–Schmidt independence criterion (HSIC) was first proposed by Gretton et al.^20^. They first map the observations into a reproducing kernel Hilbert space $\mathcal {F}$ (RKHS) generated by a given kernel $k(\cdot ,\cdot )$, that is, for each point $x\in X$, there corresponds an element (feature map) $\phi (x)\in \mathcal {F}$ such that $<\phi (x), \phi (x')>_{\mathcal {F}} = k(x,x')$, where $k: X\times X \rightarrow \mathcal {R}$ is a unique positive definite kernel. They then consider a cross-covariance operator between feature maps and the squared Hilbert–Schmidt norm of the cross-covariance operator, which can be expressed as

3 $$\begin{aligned} \text {HSIC}(f_{XY})&= E_{XX'YY'}[k_{X}(X,X')k_{Y}(Y,Y')] + E_{XX'}[k_{X}(X,X')]E_{YY'}[k_{Y}(Y,Y')]  \\&\quad -2E_{XY}\left[ E_{X'}[k_{X}(X,X')]E_{Y'}[k_{Y}(Y,Y')]\right] , \end{aligned}$$

where $X'$ and $Y'$ are independent copies of *X* and *Y*, respectively. Here, when characteristic kernels, such as the Gaussian kernel or Laplacian kernel, are used for $k_{X}$ and $k_{Y}$, $\text {HSIC}(f_{XY}) = 0$ if and only if $f_{XY} = f_{X}f_{Y}$.

An empirical estimate of HSIC was proposed by Gretton et al.^20^:

4 $$\begin{aligned} \text {HSIC}&= \frac{1}{N^2}\sum _{i,j}^{N}k_{X}\big (X_{i},X_{j}\big )k_{Y}\big (Y_{i},Y_{j}\big ) + \frac{1}{N^4}\sum _{i,j,u,v}^{N}k_{X}\big (X_{i},X_{j}\big )k_{Y}\big (Y_{u},Y_{v}\big ) - \frac{2}{N^3}\sum _{i,j,u}^{N}k_{X}\big (X_{i},X_{j}\big )k_{Y}\big (Y_{i},Y_{u}\big ). \end{aligned}$$

Let $K_{X}$ and $K_{Y}$ be kernel matrices with entries $k_{X}(X_{i},X_{j})$ and $k_{Y}(Y_{i},Y_{j})$, respectively. Then, HSIC can be rewritten as

5 $$\begin{aligned} \text {HSIC} = \frac{trace\left( \tilde{K}_{X}\tilde{K}_{Y}\right) }{N^2}, \end{aligned}$$

where $\tilde{K}_{X} = H_{N}K_{X}H_{N}$ and $\tilde{K}_{Y} = H_{N}K_{Y}H_{N}$ are the centered kernel matrices of $K_{X}$ and $K_{Y}$, respectively, and $H_{N} = I_{N} - 1_{N}1_{N}^{t}/N$ is a centering matrix with $I_{N}$ being an identity matrix of order *N* and $1_{N}$ being a $N\times 1$ vector of all ones.

Gretton et al.^21^ studied asymptotic behaviors of HSIC and found that HSIC is degenerate under the null hypothesis of independence. Hence, they proposed a few approaches to approximate it: a Gamma approximation and a permutation approach. Despite the large computational cost, they recommend the permutation approach since the Gamma approximation easily loses power due to a very low variance estimate.

### Related works

The HSIC-based test is widely used in many applications since it is powerful and versatile without strong model assumptions and the new test is also in line with this principle. Recently, several approaches have been proposed. For example, Zhan et al.^13^ proposed a kernel RV coefficient (KRV) to capture the dependence between two random variables. KRV is a generalized RV coefficient using kernels and it can capture complex relationships, such as nonlinear correlations, among the individual data types. KRV is equivalent to the new test under the permutation null distribution, but the new test has simpler forms since it does not require the standardization.

Recently, Liu et al.^27^ proposed the HSIC-based test for cluster-correlated data, denoted by $\text {HSIC}_{cl}$. They derived the asymptotic distribution of HSIC under the null hypothesis of independence between two variables but in the presence of sample correlations. Compared to the HSIC that has an inflated type I error under the cluster-correlated structure, $\text {HSIC}_{cl}$ not only controls the type I error well but also performs better than the HSIC. The asymptotic null distribution of $\text {HSIC}_{cl}$ is the mixture of chi-square distributions, but the weights are unknown and it should be estimated with empirical counterparts. A Davies’ exact method^28^ is a way to approximate the asymptotic distribution of HSIC, so the authors adopt this approach. However, the asymptotic null distribution of $\text {HSIC}_{cl}$ has more complicated expressions of the weigths than the HSIC and it needs to compute the eigenvalues of a $N^2$ by $m^2$ matrix, which provides excessive computational burden for large datasets. Moreover, the Davies’ method shows too much conservativeness (see Table 1). To address this, we work under the permutation null distribution and develop a test statistic in a simple manner. Details are in the following section.

### HSIC for cluster-correlated data

As discussed in the previous section, given a test statistic, the next question is to determine the critical value of the test with the correct size. The main challenge of the HSIC application is to determine the critical value of the test with the correct size. When using the original HSIC, a major difficulty arises in this step since the asymptotic null distribution of the HSIC is an infinite weighted sum of chi-square random variables and it cannot be applied in practice. Though the Davies’ method can be used, as discussed in the previous section, it is computationally expensive and too conservative. Moreover, this is not accurate under the small sample size setting.

To address this, we work under the permutation null distribution and determine whether to reject the null hypothesis or not by the permutation test. The permutation approach does not need to resort to the estimation, asymptotic properties, or any underlying conditions. Hence, the permutation test has been utilized in many applications^29^ and the exact cutoff for the test can be obtained from the permutation null distribution. Through *N*! permutations of shuffling rows and columns of one kernel matrix, the *p* value can be obtained as the proportion of permuted statistic values greater than or equal to the actual test statistic value. This yields a valid level of the test for finite samples.

Based on the method of obtaining the critical value of the new test under the clustered data setting in the previous section, we now consider testing the null hypothesis of independence $\text {H}_{0}: X\perp Y$. As discussed in the previous section, the HSIC is the cross-covariance operator in RKHS, but it also can be interpreted as a Euclidean-like distance measure between kernel values under the permutation distribution. To be more specific, the Euclidean-like distance measure between kernel values can be defined as follows:

$$\begin{aligned} \sum _{i,j}\left( k_{ij}^{X} - k_{ij}^{Y}\right) ^2, \end{aligned}$$

where $k_{ij}^{X}$ and $k_{ij}^{Y}$ are (*i*, *j*)-th elements of the kernel matrices $K_{X}$ and $K_{y}$, respectively. Then,

$$\begin{aligned} \sum _{i,j}\left( k_{ij}^{X} - k_{ij}^{Y}\right) ^2&= \sum _{i,j}\left( k_{ij}^{X}\right) ^2 + \sum _{i,j}\left( k_{ij}^{Y}\right) ^2 - 2\sum _{i,j}k_{ij}^{X}k_{ij}^{Y} = C - 2\sum _{i,j}k_{ij}^{X}k_{ij}^{Y} = C - 2trace\left( K_{X}K_{Y}\right) , \end{aligned}$$

where *C* is a constant under the permutation. When the kernel matrices are centered, the HSIC is equivalent to the Euclidean-like distance measure between kernel values under the permutation distribution.

**Figure 1:**
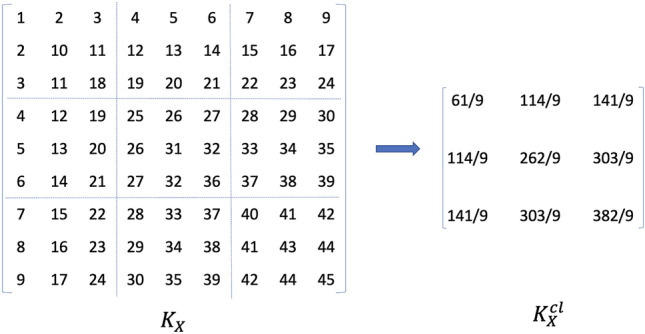
Illustration for $K_{X}^{cl}$ when $N=9$ and $m=3$.

**Figure 2:**
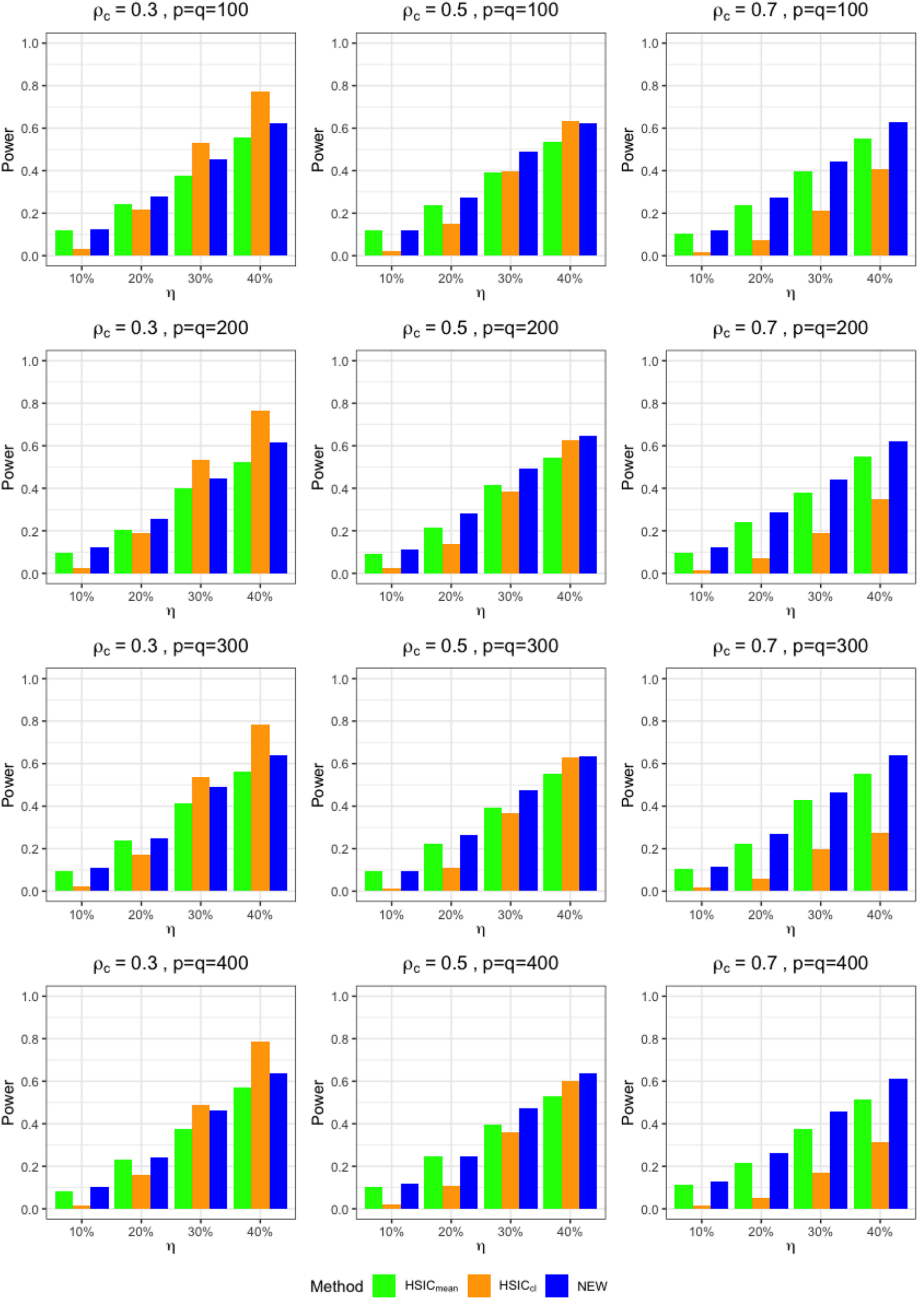
Estimated power of the tests under different exposures $(\eta )$, within-cluster correlations $(\rho _{c})$, and dimensions $p=q$ when $m=100$.

One simple way to accommodate cluster-correlated structure is to analyze data at the cluster/subject level, such as utilizing averaged observations at different clusters. However, this could result in loss of information. Moreover, variations across clusters may not be reflected (see Table 3, Fig. 2) To accommodate both differences between kernel values and variations across clusters, we define cluster-wise kernel matrices $K_{X}^{cl}$ and $K_{Y}^{cl}$. Specifically, we combine kernel information for each cluster by averaging kernel values so that the similarity within and between clusters is well reflected. In other words, the original $N\times N$ kernel matrices $K_{X}$ and $K_{Y}$ become $m\times m$ cluster-wise kernel matrices $K_{X}^{cl}$ and $K_{Y}^{cl}$, respectively. Note that $K_{X}^{cl}$ and $K_{Y}^{cl}$ are still symmetric and positive semi-definite. Figure 1 illustrates the formulation of the cluster-wise kernel matrix.

Let $\tilde{K}_{X}^{cl} = H_{m}K_{X}^{cl}H_{m}$ and $\tilde{K}_{Y}^{cl} = H_{m}K_{Y}^{cl}H_{m}$ be centered cluster-wise kernel matrices where $H_{m} = I_{m} - 1_{m}1_{m}^{t}/m$ is a centering matrix. Then, a new HSIC under the clustered data setting is defined as

6 $$\begin{aligned} \text {HSIC}_{new} = tr\left( \tilde{K}_{X}^{cl}\tilde{K}_{Y}^{cl}\right) . \end{aligned}$$

For small or moderate sample sizes, we can conduct the permutation test directly and this provides a valid level of the test. However, permutations may be computationally cumbersome when the number of clusters is large. Hence, when the number of clusters is very large, we need to estimate the permutation null distribution of the test statistic. To estimate the *p* value of the test based on $\text {HSIC}_{new}$ without permutations, we propose a moment matching approach using a Pearson type III approximation^30,31^. Specifically, we approximate the permutation null distribution of $\text {HSIC}_{new}$ by the Pearson type III distribution. This requires the first three moments of the exact permutation distribution of $\text {HSIC}_{new}$. Let $\mu$, $\sigma ^2$, and $\gamma$ be the mean, variance, and skewness of $\text {HSIC}_{new}$ obtained from the permutation null distribution Detail expressions are provided in Supplementary A). Then, the *p* value of the $\text {HSIC}_{new}$ can be analytically computed by the Pearson type III distribution

$$\begin{aligned} f(x) = \frac{1}{|s|^a\Gamma (a)}|x-\lambda |^{a-1}\exp \left( -\frac{x-\lambda }{s}\right) , \end{aligned}$$

where $a = 4/\gamma ^2$, $s = \sigma \gamma /2$, and $\lambda = \mu - 2\sigma /\gamma$. We check the efficacy of this approach and it is provided in the following “Results” section.

The choice of kernel and the bandwidth parameter have been studied for two-sample comparison. For example, Gretton et al.^32^ studied a linear combination of Gaussian kernels to maximize the power of the test and Ramdas et al.^33^ found that, under some conditions, the power of the test based on the Gaussian kernel is independent of the bandwidth when the median heuristic, the median of all pairwise distances among observations, is used. Song and Chen^34^ studied the bandwidth choice under the permutation null distribution and showed that the median heuristic is a reasonable choice. Therefore, since the main data variation is well captured by the median heuristic under the permutation null distribution, we propose to use the Gaussian kernel with the median heuristic for the proposed test.

## Results

### Efficacy of the testing procedure

In this section, we briefly check how accurate the Pearson type III approximation defined in the previous section is compared to the permutation approach as well as the Davies’ method^28^. To this end, we observe the empirical type I error rate of the HSIC tests from 1000 simulation runs and compare the performance of the Pearson type III approximation with the permutation approach and the Davies’ method for the *p*-dimensional Gaussian and log-normal data, $N_{p}({\textbf {0}}_{p},\Sigma )$ and $\log N_{p}({\textbf {0}}_{p},\Sigma )$ with $\Sigma _{(i,j)} = 0.4^{|i-j|}$, respectively, under the independent and identically distributed (i.i.d.) setting.

Table 1 shows the empirical size of the HSIC tests based on the Pearson type III approximation (Pearson), the permutation approach with 1000 permutations (Perm) and the Davies’ method (Davies) under different sample sizes and dimensions. We see that the permutation distribution can be well approximated by the Pearson type III approximation and the Pearson type III approximation in general controls the type I error well, while the Davies’ method is too conservative.

**Table 1:** Empirical size of the tests under different number of samples (*N*) and dimensions $(p=q)$ at 0.05 significance level.

Type	*N*	$p=q$	Pearson	Perm	Davies
Normal	50	50	0.049	0.050	0.000
		100	0.050	0.051	0.000
		200	0.055	0.061	0.000
	100	50	0.048	0.053	0.005
		100	0.049	0.050	0.000
		200	0.058	0.057	0.000
	200	50	0.048	0.050	0.016
		100	0.052	0.053	0.006
		200	0.053	0.053	0.001
Log-normal	50	50	0.055	0.056	0.024
		100	0.049	0.047	0.009
		200	0.041	0.040	0.005
	100	50	0.053	0.050	0.041
		100	0.052	0.053	0.038
		200	0.050	0.050	0.017
	200	50	0.054	0.054	0.058
		100	0.044	0.044	0.047
		200	0.055	0.055	0.036

We also check how much faster the Pearson type III approximation is compared to the permutation approach with 1000 permutations (perm=1000) and 10,000 permutations (perm=10,000). Notice that the permutation approach becomes more accurate as the number of permutations increases, which increases the computational time as well. Table 2 shows average runtimes for each sample size when $p=q = 100$. In comparison to the permutation approach, we see that the Pearson type III approximation can save a significant amount of computational cost.

**Table 2:** Average runtimes in seconds from 10 simulations for each sample size *N*.

*N*	100	500	1000	2000
Permutation approach (perm=1000)	0.146	4.116	17.22	78.63
Permutation approach (perm=10,000)	1.582	26.03	106.95	465.78
Pearson approximation	0.102	0.986	7.408	47.89

All experiments were run by $\texttt {R}$ on 2.2 GHz Intel Core i7.

### Power analysis

We now examine the performance of the new test through simulations. We compare the new test with the existing HSIC-based test for cluster-correlated data proposed by Liu et al.^27^, denoted by $\text {HSIC}_{cl}$, and the original HSIC. Here, we follow the simulation setup in Liu et al.^27^ for power comparison. In addition, we check the computational cost of the tests.

Specifically, we generate *m* clusters from the *p*-dimensional $(p=q)$ Gaussian data: $N_{3p}(5\times 1_{3p}, \Sigma _{X})$, where $\Sigma _{X} = \Sigma _{W}\bigotimes \Sigma _{c}$ with

$$\begin{aligned} \Sigma _{W} = \begin{pmatrix} 1 & \rho _{W} & \cdots & \rho _{W} \\ \rho _{W} & 1 & \cdots & \rho _{W} \\ \vdots & \vdots & \ddots & \vdots \\ \rho _{W} & \rho _{W} & \cdots & 1 \end{pmatrix}_{p\times p}, \ \Sigma _{c} = \begin{pmatrix} 1 & \rho _{c} & \rho _{c}^2 \\ \rho _{c} & 1 & \rho _{c} \\ \rho _{c}^2 & \rho _{c} & 1 \end{pmatrix}_{3\times 3}, \end{aligned}$$

where $\bigotimes$ is the Kronecker product. Here, we fix the cluster size 3 for all $i=1,\ldots ,m$. We also consider an exchangeable correlation structure $\Sigma _{W}$ across *p* variables in *X* and AR(1) correlation structure $\Sigma _{c}$ across three time points.

We use the Gaussian kernel and the median heuristic bandwidth. We simulate 1000 datasets and the significance level is set to be 0.05.

**Table 3:** Empirical size of the tests at 0.05 significance level under different dimensions $(p=q)$ and within-cluster correlations $(\rho _{c})$.

$p=q$	$\rho _{c}$	HSIC	$\text {HSIC}_{cl}$	$\text {HSIC}_{new}$
100	0.3	0.114	0.001	0.050
	0.5	0.821	0.000	0.045
	0.7	1.000	0.003	0.048
200	0.3	0.107	0.002	0.039
	0.5	0.893	0.004	0.046
	0.7	1.000	0.001	0.045
300	0.3	0.127	0.003	0.053
	0.5	0.912	0.002	0.054
	0.7	1.000	0.002	0.047
400	0.3	0.126	0.000	0.043
	0.5	0.919	0.002	0.040
	0.7	1.000	0.003	0.049

Table 3 shows the empirical size of tests at 0.05 significance level by 1000 simulation runs under different dimensions $(p=q)$ and within-cluster correlations $(\rho _{c})$ when $m=100$ and $\rho _{W}=0.5$. Corresponding standard errors are provided in Supplementary D. We see that the original HSIC does not control the type I error at all and the inflation increases as the within-cluster correlation increases. $\text {HSIC}_{cl}$ is too conservative. In contrast, $\text {HSIC}_{new}$ controls type I error well.

To compare the power of the tests, we choose one exposure variable from *X* at random as the causal exposure, and make the first $\eta$ proportion of the outcomes in *Y* depend on the exposure. Specifically, within each cluster, outcomes in *Y* are generated as follows: a single exposure (say, the *r*-th variable in *X*) affects multiple outcomes,

$$\begin{aligned} \left( Y_{11},Y_{12},Y_{13}, \ldots , Y_{p1},Y_{p2},Y_{p3}\right) ^{t} = \left( \beta _{1}X_{r1},\beta _{1}X_{r2},\beta _{1}X_{r3}, \ldots , \beta _{p}X_{r1},\beta _{p}X_{r2},\beta _{p}X_{r3}\right) ^{t} + \epsilon , \end{aligned}$$

where $\epsilon \sim N_{3p}({\textbf {0}}_{3p},\Sigma _{X})$ and the effect sizes $\beta _{s}$’s are generated from a Uniform$(0,\sqrt{25/m})$ $(s=1,\ldots ,\eta p)$.

In addition to $\text {HSIC}_{cl}$, we consider another HSIC-based test statistic $\text {HSIC}_{mean}$, the original HSIC test with averaged observations at different time points for each cluster, i.e., $(\sum _{j=1}^{3}X_{1j}/3,\ldots ,\sum _{j=1}^{3}X_{1p}/3)^{t}$ and $(\sum _{j=1}^{3}Y_{1j}/3,\ldots ,\sum _{j=1}^{3}Y_{1p}/3)^{t}$.

The estimated power are presented in Fig. 2. Corresponding standard errors are provided in Supplementary D. We see that the new test outperforms $\text {HSIC}_{mean}$ in all cases. $\text {HSIC}_{cl}$ shows better performance than the new test when $\rho _{c}=0.3$, but the performance decreases as $\rho _{c}$ increases. This is expected since averaging data at the cluster level will result in reduced evident information loss under high within-cluster correlation, while the new test keeps using the kernel information and still captures this signal. In addition, the new test works well under high dimensions.

In addition, we conduct power comparison between the new test and $\text {HSIC}_{mean}$ by the permutation test, Pearson type III approximation, and Davies’ method, and results are provided in Supplementary B.

We also compare the computational cost of the new test with $\text {HSIC}_{mean}$ and $\text {HSIC}_{cl}$ and the results are shown in Table 4. We see that the new test is much faster than $\text {HSIC}_{cl}$ with good performance. $\text {HSIC}_{mean}$ is the fastest, but it has lower power than the new test.

**Table 4:** Average runtimes in seconds from 10 simulations for each number of clusters *m*.

*m*	100	200	300	400
$\text {HSIC}_{mean}$	0.092	0.428	1.144	2.38
$\text {HSIC}_{cl}$	1.568	21.21	99.89	306.20
$\text {HSIC}_{new}$	0.130	0.864	7.671	22.83

All experiments were run by $\texttt {R}$ on 2.2 GHz Intel Core i7.

Lastly, we also compare the performance of the new test to other existing independence tests, dCov^14^ and HHG^35^ that are based on the distance covariance and ranking of interpoint distances, respectively, and results are provided in Supplementary C.

### Analysis of MsFLASH data

As noted previously, the MsFLASH study was a randomized study of vaginal estrogen vs. two different placebos. To understand why the trial was negative, investigators were interested in studying whether microbiome is associated with metabolic pathways. Vaginal microbiota and vaginal fluid metabolites were characterized longitudinally and available in 141 participants^36^. For each arm, we have 45, 46, and 50 clusters (corresponding to a separate subject) with the equal cluster size 3 (corresponding to three clinical visits in which vaginal swabs were obtained). The vaginal microbiome profiles include abundance data of 381 taxa. The metabolome profiles comprise the abundance data of 171 metabolites that are grouped into 95 metabolic pathways. Across all 95 pathways, we conduct the association tests to detect the dependence between metabolites in each pathway and the overall vaginal microbiome compositions.

Here, we use the Gaussian kernel as well as the Bray–Curtis kernel that can be useful when the phylogenetic tree information is unavailable. For each test, the Bonferroni-corrected significance level is set to be $0.05/95 = 5.3\times 10^{-4}$. Table 5 shows the number of detected metabolic pathways associated with the vaginal microbiome composition. We see that the new method identifies a larger number of pathways than $\text {HSIC}_{mean}$ and $\text {HSIC}_{cl}$ for all cases, indicating the consistent improvement of the new test. In particular, the new test using the Gaussian kernel is more powerful than the Bray–Curtis kernel, indicating a possible non-linear relationship between some metabolites and microbial taxa abundances.

Collectively, these results indicate that for many key biological pathways, the link between the microbiota and metabolome remains in place and as expected. Thus, the failure of the MsFLASH trial may not result from a failure in this part of the hypothesis and additional work is needed to understand why the trial failed.

**Table 5:** The number of detected metabolic pathways associated with the vaginal microbiome composition.

Gaussian	$\text {HSIC}_{mean}$	$\text {HSIC}_{cl}$	$\text {HSIC}_{new}$
Arm 1	10	8	27
Arm 2	14	4	33
Arm 3	9	4	29

Bray–Curtis	$\text {HSIC}_{mean}$	$\text {HSIC}_{cl}$	$\text {HSIC}_{new}$
Arm 1	5	8	21
Arm 2	14	8	35
Arm 3	9	3	29

## Discussion

We have introduced the new kernel-based test of independence for cluster-correlated data. The new approach is versatile and robust in that it avoids any parametric assumptions or settings. We have also proposed the analytic formulas for type I error control, offering easy off-the-shelf tools for large datasets. We have experimentally demonstrated that the new test exhibits superior power and work well particularly for high-dimensional settings with large within-cluster correlation.

As demonstrated, our approach is effective in assessing the generalized dependency between two sets of data when the samples are clustered. However, while our approach accommodates the correlation arising from the fact that multiple samples come from the same individual, we do not explicitly harness the longitudinal nature. Specifically, we primarily treat the samples as repeated measurements rather than true longitudinal profiles in assessing association. How to bring in the longitudinal structure remains a question of importance and a topic for further investigation.

Our approach begins with pre-constructed kernel measures capturing pair-wise similarity in samples and is valid for any positive definite kernel metrics. However, kernel metrics that better capture the true relationship between the data will lead to improved power. Choosing an optimal kernel represents a general problem within the statistical learning literature. Some have proposed omnibus tests based on weighted averages of kernels, but this is a sub-optimal strategy since the HSIC statistics depend on the scale of the different kernels. A better solution is to move from the HSIC statistic to the *p* value scale with incorporation of permutation testing. However, this is again slow. One potential solution is to use the Cauchy-Combination method within this context^37^, but further evaluation is necessary.

A major contribution of this work is the computational efficiency of the proposed strategy which generalizes to both clustered and un-clustered data settings. The use of the Pearson type III approximation of the finite sample permutation distribution is fast which allows for accurate computation of tailed *p* values. For example, in an mbGWAS study looking at relationship between groups of SNPs and microbiome composition^38^, it is necessary to compute tens of thousands of tests and get *p* values at alpha levels as low as $10^{-6}$, for which permutation is infeasible. Consequently, the relevance and importance of our strategy will only continue to grow as such studies become more common.

## Supplementary Information

Supplementary Information.

## Data Availability

The data generated during and/or analyzed during the current study are available from the corresponding author on reasonable request.

## References

[1] McMillan A (2015). A multi-platform metabolomics approach identifies highly specific biomarkers of bacterial diversity in the vagina of pregnant and non-pregnant women. Sci. Rep..

[2] Liu Y, Hou Y, Wang G, Zheng X, Hao H (2020). Gut microbial metabolites of aromatic amino acids as signals in host-microbe interplay. Trends Endocrinol. Metab..

[3] Muller E, Algavi YM, Borenstein E (2021). A meta-analysis study of the robustness and universality of gut microbiome-metabolome associations. Microbiome.

[4] Mick E (2010). Family-based genome-wide association scan of attention-deficit/hyperactivity disorder. J. Am. Acad. Child Adolesc. Psychiatry.

[5] Zeger SL, Irizarry R, Peng RD (2006). On time series analysis of public health and biomedical data. Annu. Rev. Public Health.

[6] Mitchell CM (2018). Efficacy of vaginal estradiol or vaginal moisturizer vs placebo for treating postmenopausal vulvovaginal symptoms: A randomized clinical trial. JAMA Intern. Med..

[7] Pearson K (1895). Notes on regression and inheritance in the case of two parents. Proc. R. Soc. Lond..

[8] Kendall MG (1938). A new measure of rank correlation. Biometrika.

[9] Spearman C (1987). The proof and measurement of association between two things. Am. J. Psychol..

[10] Smilde AK, Kiers HA, Bijlsma S, Rubingh C, Van Erk M (2009). Matrix correlations for high-dimensional data: The modified RV-coefficient. Bioinformatics.

[11] Mayer C-D, Lorent J, Horgan GW (2011). Exploratory analysis of multiple omics datasets using the adjusted RV coefficient. Stat. Appl. Genet. Mol. Biol..

[12] Minas C, Curry E, Montana G (2013). A distance-based test of association between paired heterogeneous genomic data. Bioinformatics.

[13] Zhan X, Plantinga A, Zhao N, Wu MC (2017). A fast small-sample kernel independence test for microbiome community-level association analysis. Biometrics.

[14] Székely GJ, Rizzo ML, Bakirov NK (2007). Measuring and testing dependence by correlation of distances. Ann. Stat..

[15] Székely GJ, Rizzo ML (2013). The distance correlation t-test of independence in high dimension. J. Multivar. Anal..

[16] Lyons R (2013). Distance covariance in metric spaces. Ann. Probab..

[17] Friedman JH, Rafsky LC (1983). Graph-theoretic measures of multivariate association and prediction. Ann. Stat..

[18] Heller R, Gorfine M, Heller Y (2012). A class of multivariate distribution-free tests of independence based on graphs. J. Stat. Plan. Inference.

[19] Moon H, Chen K (2020). Interpoint-ranking sign covariance for test of independence. Biometrika.

[20] Gretton, A., Bousquet, O., Smola, A. & Schölkopf, B. Measuring statistical dependence with Hilbert–Schmidt norms. In *International Conference on Algorithmic Learning Theory*, 63–77 (Springer, 2005).

[21] Gretton, A. *et al.* A kernel statistical test of independence. In *Nips*, vol. 20, 585–592 (Citeseer, 2007).

[22] Zhao N (2015). Testing in microbiome-profiling studies with MiRKAT, the microbiome regression-based kernel association test. Am. J. Hum. Genet..

[23] Zhan X (2017). A small-sample multivariate kernel machine test for microbiome association studies. Genet. Epidemiol..

[24] Zhao N (2018). Kernel machine methods for integrative analysis of genome-wide methylation and genotyping studies. Genet. Epidemiol..

[25] Lozupone C, Knight R (2005). UniFrac: A new phylogenetic method for comparing microbial communities. Appl. Environ. Microbiol..

[26] Chen J (2012). Associating microbiome composition with environmental covariates using generalized UniFrac distances. Bioinformatics.

[27] Liu H, Plantinga A, Xiang Y, Wu M (2021). A kernel-based test of independence for cluster-correlated data. Adv. Neural Inf. Process. Syst..

[28] Davies RB (1980). The distribution of a linear combination of $$\chi $$2 random variables. J. R. Stat. Soc.: Ser. C (Appl. Stat.).

[29] Good, P. *Permutation Tests: A Practical Guide to Resampling Methods for Testing Hypotheses* (Springer, 2013).

[30] Mielke, P. W. & Berry, K. J. *Permutation Methods: A Distance Function Approach* (Springer, 2007).

[31] Josse J, Pagès J, Husson F (2008). Testing the significance of the RV coefficient. Comput. Stat. Data Anal..

[32] Gretton, A. *et al.* Optimal kernel choice for large-scale two-sample tests. In *Advances in Neural Information Processing Systems*, 1205–1213 (Citeseer, 2012).

[33] Ramdas, A., Reddi, S. J., Poczos, B., Singh, A. & Wasserman, L. Adaptivity and computation-statistics tradeoffs for kernel and distance based high dimensional two sample testing. arXiv:1508.00655 (2015).

[34] Song, H. & Chen, H. A fast and effective large-scale two-sample test based on kernels. arXiv:2110.03118 (2021).

[35] Heller R, Heller Y, Gorfine M (2013). A consistent multivariate test of association based on ranks of distances. Biometrika.

[36] Mitchell CM (2021). Association between postmenopausal vulvovaginal discomfort, vaginal microbiota, and mucosal inflammation. Am. J. Obstet. Gynecol..

[37] Liu Y, Xie J (2020). Cauchy combination test: A powerful test with analytic p-value calculation under arbitrary dependency structures. J. Am. Stat. Assoc..

[38] Liu, H. *et al.* Kernel-based genetic association analysis for microbiome phenotypes identifies host genetic drivers of beta-diversity. *bioRxiv* (2021). 10.1186/s40168-023-01530-0 PMC10116795 37081571

