# Supplementary material for: A fast kernel independence test for cluster-correlated data
Source: Sci Rep. 2022 Dec 15;12:21659. doi: 10.1038/s41598-022-26278-9 (PMC9755291; doi:10.1038/s41598-022-26278-9)
Supplement: Supplementary file 1 — Supplementary Information. [file 41598_2022_26278_MOESM1_ESM.pdf]

Supplementary A: Analytic expressions for the first three moments

The Pearson type III approximation for the permutation null distribution of the new test statistic requires the first three moments of HSIC$_{new}$ under the permutation null distribution. Under the permutation null distribution, the analytic formulas for the mean (μ) and the variance (σ^2) of HSIC$_{new}$ can be obtained as follows Kazi et al.[1]:

$$\mu = \frac{tr(\tilde{K}_X^{cl})tr(\tilde{K}_Y^{cl})}{m-1},$$
$$\sigma^2 = T_2^X T_2^Y \frac{2(m-1-\beta_X)(m-1-\beta_Y)}{(m+1)(m-1)^2(m-2)}\left(1+\frac{(m-3)\tau_X\tau_y}{2m(m-1)}\right),$$

where

$$T_2^X = tr((\tilde{K}_X^{cl})^2),\ S_2^X = \sum_{i=1}^{m}((\tilde{K}_X^{cl})_{ii})^2,\ \beta_X = \frac{(tr(\tilde{K}_X^{cl}))^2}{T_2^X},\ \tau_X = \frac{n-1}{(n-3)(n-1-\beta_X)}\left(\frac{n(n+1)S_2^X}{T_2^X}-(n-1)(\beta_X+2)\right),$$

and T_2^Y, S_2^Y, β_Y, and τ_Y are defined correspondingly. The skewness (γ) also can be obtained similarly. Since

$$\gamma = \frac{E(\text{HSIC}_{new}^3)-3\mu\sigma^2-\mu^3}{\sigma^3},$$

and the analytic expressions for μ and σ can be found in Materials and methods section, we only need to figure out the analytic expressions for the third moment. For the third moment, we have

$$\begin{aligned}
&m(m-1)(m-2)(m-3)(m-4)(m-5)E\left(\text{HSIC}_{new}^3\right)\\
&= m^2(m+1)(m^2+15m-4)S_3^X S_3^Y + 4(m^4-8m^3+19m^2-4m-16)U_X U_Y\\
&+24(m^2-m-4)(U_X B_Y + U_Y B_X) + 6(m^4-8m^3+21m^2-6m-24)B_X B_Y\\
&+12(m^4-m^3-8m^2+36m-48)R_X R_Y + 12(m^3-2m^2+9m-12)(T_X S_2^X R_Y + T_Y S_2^Y R_X)\\
&+3(m^4-4m^3-2m^2+9m-12)T_X T_Y S_2^X S_2^Y + 24(m^3-3m^2-2m+8)(R_X U_Y + R_Y U_X)\\
&+24(m^3-2m^2-3m+12)(R_X B_Y + R_Y B_X) + 12(m^2-m+4)(T_X S_2^X U_Y + T_Y S_2^Y U_X)\\
&+6(2m^3-7m^2-3m+12)(T_X S_2^X B_Y + T_Y S_2^Y B_X)\\
&-2m(m-1)(m^2-m+4)\{(2U_X+3B_X)S_3^Y + (2U_Y+3B_Y)S_3^X\}\\
&-3m(m-1)^2(m+4)\{(T_X S_2^X + 4R_X)S_3^Y + (T_Y S_2^Y + 4R_Y)S_3^X\}\\
&+2m(m-1)(m-2)\{(T_X^3+6T_X T_2^X + 8T_3^X)S_3^Y + (T_Y^3 + 6T_Y T_2^Y + 8T_3^Y)S_3^X\}\\
&+T_X^3\{(m^3-9m^2+23m-14)T_Y^3 + 6(m-4)T_Y T_2^Y + 8T_3^Y\}\\
&+6T_X T_2^X\{(m-4)T_Y^3 + (m^3-9m^2+24m-14)T_Y T_2^Y + 4(m-3)T_3^Y\}\\
&+8T_3^X\{T_Y^3 + 3(m-3)T_Y T_2^Y + (m^3-9m^2+26m-22)T_3^Y\}\\
&-16(T_X^3 U_Y + U_X T_Y^3) - 6(2m^2-10m+16)(T_X T_2^X U_Y + T_Y T_2^Y U_X)\\
&-8(3m^2-15m+16)(T_3^X U_Y + U_X T_3^Y) - (6m^2-30m+24)(T_X^3 B_Y + B_X T_Y^3)\\
&-6(4m^2-20m+24)(T_X T_2^X B_Y + T_Y T_2^Y B_X)\\
&-8(3m^2-15m+24)(T_3^X B_Y + T_3^Y B_X) - 24(m-2)(T_X^3 R_Y + T_Y^3 R_X)\\
&-6(m-2)(2m^2-10m+24)(T_X T_2^X R_Y + T_Y T_2^Y R_X)\\
&-8(m-2)(3m^2-15m+24)(T_3^X R_Y + T_3^Y R_X)\\
&-(m-2)(3m^2-15m+6)(T_X^3 T_Y S_2^Y + T_Y^3 T_X S_2^X) - 48(m-2)(T_3^X T_Y S_2^Y + T_3^Y T_X S_2^X)\\
&-6(m-2)(m^2-5m+6)(T_X T_2^X T_Y S_2^Y + T_Y T_2^Y T_X S_2^X),
\end{aligned}$$

where

$$T_X = tr(\tilde{K}_X^{cl}),\ \ T_2^X = tr((\tilde{K}_X^{cl})^2),\ \ T_3^X = tr((\tilde{K}_X^{cl})^3),$$

$$S_2^X = \sum_{i=1}^{m}((\tilde{K}_X^{cl})_{ii})^2,\ \ S_3^X = \sum_{i=1}^{m}((\tilde{K}_X^{cl})_{ii})^3,$$

$$U_X = \sum_{i,j=1}^{m}((\tilde{K}_X^{cl})_{ij})^3,\ \ B_X = (diag(\tilde{K}_X^{cl}))^t \tilde{K}_X^{cl} diag(\tilde{K}_X^{cl}),$$

$$R_X = (diag(\tilde{K}_X^{cl}))^t diag((\tilde{K}_X^{cl})^2).$$

Similarly, $T_Y, T_2^Y, T_3^Y, S_2^Y, S_3^Y, U_Y, B_Y, R_Y$ are obtained from the kernel matrix $\tilde{K}_Y^{cl}$.

Supplementary B: Detail comparison between $HSIC_{new}$ and $HSIC_{mean}$

In this section, we conduct detail power comparison between $HSIC_{new}$ and $HSIC_{mean}$ by 1,000 permutations, the Pearson type III approximation, and Davies' method under the simulation setting in Power analysis section in the main paper. Table S1 shows the empirical size of the tests at 0.05 significance level. We see that the Davies' method is more conservative than the permutation approach and the permutation approach can be well approximated by the Pearson type III approximation. Table S2 shows the estimated power of the tests. We see that the new test with the Pearson type III aproximation outperforms $HSIC_{mean}$ in all cases.

$p = q$	ρ_c	HSIC$_{mean}$ (Davies)	HSIC$_{mean}$ (Permu)	HSIC$_{mean}$ (Pearson)	HSIC$_{new}$ (Permu)	HSIC$_{new}$ (Pearson)
100	0.3	0.039	0.042	0.036	0.047	0.050
	0.5	0.032	0.052	0.050	0.056	0.045
	0.7	0.037	0.056	0.053	0.059	0.048
200	0.3	0.042	0.053	0.046	0.063	0.039
	0.5	0.037	0.057	0.055	0.065	0.046
	0.7	0.042	0.049	0.043	0.050	0.046

Table S1. Empirical size of the tests at 0.05 significance level under different dimensions $(p = q)$ and within-cluster correlations (ρ_c).

Supplementary C: Power comparison of the new test to dCov and HHG

In this section, we compare the performance of the new test to other existing independence tests as a simple baseline. Here, we consider popular nonparametric tests, dCov[2] and HHG[3] that are based on the distance covariance and ranking of interpoint distances, respectively. Table S3 shows the empirical size of the tests at 0.05 significance level under the simulation setting in Power analysis section in the main paper. We see that the existing independence tests, dCov and HHG, cannot control the type I error under the cluster-correlated structure. In particular, this phenomenon gets worse as the within-cluster correlations or dimensions increase.

Supplementary D: Standard errors of the results in Table 3 and Figure 2

Here, Table S4 and S5 show corresponding standard erros of the results in Table 3 and Figure 2, respectively.

$p=q$	ρ_c	η	HSIC$_{mean}$ (Davies)	HSIC$_{mean}$ (Permu)	HSIC$_{mean}$ (Pearson)	HSIC$_{new}$ (Permu)	HSIC$_{new}$ (Pearson)
100	0.3	10%	0.120	0.107	0.098	0.121	0.126
		20%	0.244	0.254	0.238	0.275	0.279
		30%	0.375	0.452	0.424	0.493	0.453
		40%	0.558	0.617	0.595	0.686	0.621
	0.5	10%	0.117	0.118	0.111	0.130	0.120
		20%	0.236	0.260	0.235	0.302	0.279
		30%	0.393	0.442	0.423	0.502	0.491
		40%	0.537	0.597	0.574	0.655	0.622
	0.7	10%	0.103	0.116	0.099	0.102	0.119
		20%	0.239	0.266	0.253	0.292	0.274
		30%	0.397	0.433	0.402	0.481	0.442
		40%	0.549	0.600	0.574	0.647	0.627
200	0.3	10%	0.095	0.126	0.113	0.135	0.123
		20%	0.205	0.230	0.218	0.264	0.254
		30%	0.401	0.454	0.436	0.494	0.455
		40%	0.521	0.580	0.551	0.653	0.615
	0.5	10%	0.093	0.113	0.105	0.108	0.111
		20%	0.214	0.254	0.234	0.271	0.282
		30%	0.416	0.439	0.419	0.499	0.490
		40%	0.543	0.598	0.569	0.663	0.649
	0.7	10%	0.098	0.112	0.104	0.116	0.121
		20%	0.239	0.243	0.231	0.261	0.285
		30%	0.377	0.415	0.387	0.442	0.442
		40%	0.550	0.596	0.571	0.657	0.620

Table S2. Estimated power of the tests under different exposures (η), within-cluster correlations (ρ_c), and dimensions $p=q$ when $m=100$.

$p=q$	ρ_c	dCov	HHG	HSIC$_{new}$
100	0.3	0.088	0.066	0.050
	0.5	0.310	0.149	0.045
	0.7	1.000	0.531	0.048
200	0.3	0.105	0.063	0.039
	0.5	0.343	0.150	0.046
	0.7	1.000	0.555	0.045

Table S3. Empirical size of the tests at 0.05 significance level under different dimensions ($p=q$) and within-cluster correlations (ρ_c).

$p=q$	ρ_c	HSIC	HSIC$_{cl}$	HSIC$_{new}$
100	0.3	0.002	0.000	0.001
	0.5	0.001	0.000	0.001
	0.7	0.000	0.000	0.001
200	0.3	0.004	0.000	0.002
	0.5	0.003	0.000	0.001
	0.7	0.000	0.001	0.001
300	0.3	0.004	0.003	0.002
	0.5	0.003	0.000	0.001
	0.7	0.000	0.001	0.001
400	0.3	0.004	0.002	0.003
	0.5	0.003	0.001	0.002
	0.7	0.000	0.001	0.001

Table S4. Corresponding standard errors of the results in Table 3.

$p=q$	ρ_c	η	HSIC	HSIC_{cl}	HSIC_{new}
100	0.3	10%	0.002	0.002	0.003
		20%	0.003	0.003	0.004
		30%	0.005	0.004	0.005
		40%	0.005	0.005	0.004
	0.5	10%	0.003	0.001	0.001
		20%	0.004	0.003	0.002
		30%	0.006	0.005	0.005
		40%	0.003	0.002	0.004
	0.7	10%	0.002	0.001	0.003
		20%	0.005	0.003	0.055
		30%	0.002	0.004	0.003
		40%	0.005	0.003	0.004
200	0.3	10%	0.003	0.001	0.002
		20%	0.003	0.002	0.004
		30%	0.004	0.006	0.004
		40%	0.003	0.004	0.005
	0.5	10%	0.004	0.001	0.003
		20%	0.002	0.003	0.003
		30%	0.003	0.004	0.004
		40%	0.003	0.005	0.005
	0.7	10%	0.002	0.001	0.002
		20%	0.007	0.003	0.004
		30%	0.004	0.002	0.005
		40%	0.007	0.005	0.004
300	0.3	10%	0.002	0.001	0.002
		20%	0.002	0.002	0.003
		30%	0.003	0.001	0.002
		40%	0.003	0.002	0.003
	0.5	10%	0.004	0.002	0.003
		20%	0.004	0.004	0.004
		30%	0.004	0.004	0.004
		40%	0.004	0.004	0.004
	0.7	10%	0.004	0.002	0.004
		20%	0.005	0.003	0.005
		30%	0.004	0.003	0.005
		40%	0.004	0.003	0.005
400	0.3	10%	0.003	0.003	0.003
		20%	0.004	0.003	0.004
		30%	0.004	0.003	0.003
		40%	0.004	0.003	0.004
	0.5	10%	0.004	0.001	0.003
		20%	0.003	0.003	0.004
		30%	0.003	0.003	0.005
		40%	0.004	0.004	0.005
	0.7	10%	0.003	0.002	0.004
		20%	0.005	0.003	0.005
		30%	0.004	0.005	0.004
		40%	0.006	0.004	0.005

Table S5. Corresponding standard errors of the results in Figure 2.

References

1. Kazi-Aoual, F., Hitier, S., Sabatier, R. & Lebreton, J.-D. Refined approximations to permutation tests for multivariate inference. *Comput. statistics & data analysis* **20**, 643–656 (1995).

2. Székely, G. J., Rizzo, M. L. & Bakirov, N. K. Measuring and testing dependence by correlation of distances. *The annals statistics* **35**, 2769–2794 (2007).

3. Heller, R., Heller, Y. & Gorfine, M. A consistent multivariate test of association based on ranks of distances. *Biometrika* **100**, 503–510 (2013).
